# Acute Pancreatitis: Clinical Profile of 60 Patients

**DOI:** 10.7759/cureus.51234

**Published:** 2023-12-28

**Authors:** Sukhwinder Thakur, Rupinderjeet Kaur, Lovleen Bhatia, Richa Bansal, Ardaman Singh, Jaskaran Singh

**Affiliations:** 1 Department of Medicine, Government Medical College, Patiala, Patiala, IND

**Keywords:** alcohol, serum lipase, multi organ dysfunction syndrome, pancreatic necrosis, acute pancreatitis

## Abstract

Introduction

Acute pancreatitis (AP) is a common differential diagnosis of acute pain abdomen and cannot be considered self-limiting as it has serious early and long-term impacts. Depending on severity, AP is divided into mild, moderately severe, and severe AP. Management of AP involves accurate diagnosis, high-quality supportive care, monitoring for early detection and treatment of complications, and prevention of relapse.

Aim

To assess the etiological risk factors, clinical profile, and complications in patients with AP.

Methods

The present study was conducted on 60 eligible patients admitted to the Department of Medicine, Government Medical College, and Hospital of Northern India. A diagnosis of AP was established based on the revised Atlanta classification (2012) for the classification of AP, and relevant data were collected and statistically analyzed.

Results

Most of the AP patients were in the 21-40 year age group. The majority were males (88.3%). Alcohol was the most common etiological factor in 76.7% of patients followed by cholelithiasis in 10% of patients. Pain abdomen was the most common presenting clinical feature occurring in 96.7% of patients and vomiting in 65% of the patients. Acute fluid collection was the most common pancreatic complication occurring in 26.7% of the patients, pancreatic edema was seen in 21.7%, and pancreatic necrosis in 15%. Among extrapancreatic complications, ascites was most commonly seen in 50% of patients followed by pleural effusion in 15%, shock in 15%, multiple organ dysfunction syndrome (MODS) in 15%, and hypocalcemia in 11.7% of patients.

Conclusion

AP should be one of the differentials for patients presenting with pain abdomen, especially when probable risk factors such as alcohol abuse and cholelithiasis are present. A high index of suspicion to diagnose AP is needed as timely management may prevent systematic complications, thus improving the outcome. Poor prognostic indicators are raised levels of total serum bilirubin, raised serum lipase, reduced serum albumin, and low platelet count among AP patients.

## Introduction

Acute pancreatitis (AP) is a common inflammatory disease of the exocrine pancreas that cannot be considered self-limiting, as it has serious early and long-term impacts. With a global incidence of 30-40 cases per 100,000 people per year, the mortality rate ranges from 1% to 5% [1]. AP is characterized by autodigestion of the gland by pancreatic digestive enzymes. Injury to the pancreas leads to the production and liberation of proinflammatory cytokines, chemokines, and other biologically active compounds. The splanchnic area is the primary site of inflammatory mediators’ release, and their access to the systemic compartment is mainly by the lymphatic, portal vein, and suprahepatic circulation. Gut barrier failure, with bacteria and endotoxin translocation, has been proposed as a major contributor to the development of local infection and multi-organ failure in severe AP (SAP). Intestinal permeability disturbances have been found in SAP patients 72 hours after onset, and they correlate strongly with clinical outcomes [2].

AP can be subdivided into two types: (a) interstitial oedematous pancreatitis and (b) necrotizing pancreatitis. Depending on the severity, AP is divided into mild AP (no organ failure, no local or systemic complications), moderately severe AP (organ failure that resolves within 48 hours, i.e., transient organ failure, and/or local or systemic complications without persistent organ failure), and severe AP (persistent organ failure for more than 48 hours, that may involve single or multiple organs) [3]. Common causes of pancreatitis include gallstones, alcohol, hypertriglyceridemia, drugs, post-endoscopic retrograde cholangiopancreatography (post-ERCP), and postsurgery.

Management of AP involves accurate diagnosis, high-quality supportive care, monitoring for early detection and treatment of complications, and prevention of relapse. Adequate prompt fluid resuscitation is crucial in the prevention of systemic complications [4].

Because of the frequent emergency, multimodality presentation, and various complications, this challenging subject is taken for the present study, which aims at understanding the etiology, clinical profile, and complications associated with AP. Additionally, this study was planned to identify different risk factors/parameters associated with pancreatic/extra-pancreatic complications that can be used to monitor and predict the occurrence of a particular complication and remediable factors that, if corrected, can improve the progression of the disease.

## Materials and methods

The objective of the study was to determine the etiological risk factors, clinical profile, and complications in patients with AP. 

Sample size calculation

As per our hospital’s preliminary estimates, the approximate prevalence of AP in our medicine department is 9%, with a confidence level of 99% and a precision of 10%.

Using formula

n = Z^2^P (1-P)/d^2^

n = 2.58^2^ x 0.09 (1-0.09)/0.1^2^ = 54.5

n = sample size

Z = confidence level

P = expected prevalence

d = precision

Thus, the calculated sample size was 55.

Inclusion criteria

(1) Patients of either sex with age ≥20 years.

(2) Patients presenting with acute abdominal pain associated with raised serum amylase/lipase and/or with swollen pancreatic parenchyma detected by USG/CT scan.

Exclusion criteria

(1) Patients with chronic pancreatitis.

(2) Patients not consenting for enrollment in the study.

Ethical clearance for the study was obtained from the Institutional Ethical Committee (Government Medical College, Patiala; approval no. 8(109)2019/12566, dated: 23.06.2020). Then, after taking written informed consent, 60 eligible patients admitted to the Department of Medicine, Government Medical College, and Hospital of Northern India were enrolled for the study. This was a hospital-based cross-sectional observational study, conducted from May 2020 to August 2021. Laboratory investigations: serum amylase, lipase, renal function tests, blood sugar, serum triglycerides, serum calcium, and hemogram were performed, and all the values were recorded and analyzed. Ultrasonography (USG) and X-ray abdomen were conducted in each case as the first radiological investigation, followed by a CT scan abdomen, if needed. Diagnosis of AP was established based on the revised Atlanta classification (2012) for the classification of AP [3], which includes fulfilling two of the following three criteria: a) acute onset, severe abdominal pain consistent with AP; b) three-fold increase in serum amylase or serum lipase levels of upper limit of normal; and c) radiological investigations (i.e., USG abdomen or CT scan abdomen or magnetic resonance imaging (MRI) abdomen showing evidence of AP). Data were processed and reported in terms of mean, standard deviation, and percentages. Appropriate statistical tests of comparison were applied. Statistical significance was taken as p<0.05. Data were analyzed using Statistical Product and Service Solutions (SPSS version 22; IBM SPSS Statistics for Windows, Armonk, NY) and Microsoft Excel.

## Results

We have observed male preponderance with a male:female ratio of 7.5:1. The most common age group presentation was 21-40 years, with 70% of the patients in this age group (Table 1).

**Table 1:** Gender wise distribution of patients among different age groups.

Age group (in years)	Male	Female	Total
21-40	40	2	42 (70%)
41-60	11	4	15 (25%)
>60	2	1	3 (5%)
Total	53	7	60 (100%)

Alcohol was the most common probable etiological factor observed in 46 (76.7%) patients, followed by cholelithiasis in six (10%) patients, drug-induced pancreatitis in one (1.7%), hypertriglyceridemia in one (1.7%), and hypercalcemia in one (1.7%) patient. No known cause could be found in five (8.3%) patients. There was a statistically significant difference (p<0.001) in the etiological factors of AP between males and females, with alcohol being the most common etiological factor among males (86.7%), and cholelithiasis being the most common among females (71.4%) in our study. Male/female gender difference was not statistically significant for other aetiologies (e.g., idiopathic, drug-induced, hypertriglyceridemia, and hypercalcemia) (Table 2).

**Table 2:** Etiology-wise distribution of acute pancreatitis patients and comparison among gender groups.

Etiology	Male	Female	Total n=60 (%)	P value
Alcohol	46	0	46 (76.7)	<0.001
Cholelithiasis	1	5	6 (10.0)	0.001
Idiopathic	3	2	5 (8.3)	0.029
Drug-induced	1	0	1 (1.7)	0.998
Hypertriglyceridemia	1	0	1 (1.7)	0.998
Hypercalcemia	1	0	1 (1.7)	0.999

Pain abdomen was the most common presenting clinical feature, as it was present in 96.7% (58/60) of the patients, followed by vomiting in 65% (39/60), jaundice in 16.7% (10/60), dyspnea in 11.7% (7/60), oliguria in 11.7% (7/60), and fever in 6.7% (4/60) of patients. There was no statistically significant difference in the clinical features of AP between males and females in this study.

Among the pancreatic complications, acute fluid collection was the most common complication observed in 26.7% (16/60) of the patients followed by pancreatic edema in 21.7% (13/60) and pancreatic necrosis in 15% (9/60) of patients. There was overlap among pancreatic complications as 8.3% (5/60) of patients had acute fluid collection and pancreatic edema, and 5% (3/60) of patients had acute fluid collection as well as pancreatic necrosis, but none had all these three pancreatic complications together. About 50% (30/60) of the patients had none of the pancreatic complications.

Among the extra-pancreatic complications, ascites was the most common complication reported in 50% (30/60) of the patients, followed by pleural effusion in 15% (9/60), shock in 15% (9/60), multiple organ dysfunction syndrome (MODS) in 15% (9/60), and hypocalcemia in 11.7% (7/60) of the patients.

In our study, we observed that the increase in serum lipase and serum bilirubin levels were significantly associated (P<0.05) with an increase in pancreatic/ extra-pancreatic complications (Table 3). In contrast, the difference in the mean values of serum calcium, urea, creatinine, and lipid profile was not statistically significant between the complications group and the no-complications group. Compared to laboratory parameters between male and female patients, no significant difference was noted in this study.

**Table 3:** Relationship of laboratory parameters and complications in patients of acute pancreatitis.

Laboratory parameter	Pancreatic and extra-pancreatic complications		No complications		P value
	Mean	SD	Mean	SD	
Hemoglobin	12.08	2.51	11.29	2.35	0.184
Total leucocyte count	12024	6108.27	10342	2972.21	0.681
Platelets	191630	93539.22	310270	186609	0.022
Blood urea	44.24	36.39	39.45	21.58	0.579
Serum creatinine	1.48	1.19	1.12	0.45	0.752
Serum bilirubin	1.9	1.76	0.87	0.72	0.024
Serum aspartate transaminase	80.67	70.87	105.64	135.6	0.76
Serum alanine transaminase	57.18	61.6	117.09	181.65	0.456
Serum alkaline phosphatase	121.61	56.39	113.64	38.12	0.916
Serum albumin	3.3	0.6	3.75	0.32	0.007
Serum globulin	3.04	0.63	3.09	0.55	0.73
Serum amylase	536.02	589.72	322.18	270.24	0.456
Serum lipase	591.96	666.3	144.45	136.07	<0.001
Serum Na^+^	135.24	5.21	136.64	4.57	0.438
Serum K^+^	4.19	0.55	4.41	0.69	0.324
Corrected Ca^++^	9.35	1.42	8.74	0.63	0.119
High-density lipoprotein	43.43	10.64	41.27	8.95	0.479
Low-density lipoprotein	99.69	35.25	112.82	38.84	0.298
Very low-density lipoprotein	37.31	9.07	31.55	12.91	0.231
Triglycerides	130.82	55.67	152	64.4	0.093

## Discussion

AP is a common differential among patients presenting with pain abdomen; characterized by local and systemic inflammation ranging from mild localized disease to severe systemic inflammatory disease, MODS, and death.

The male preponderance was observed in our study, with 88.3% of the study population being male. This is comparable to the studies done by Ramu et al. [5] and Negi et al. [6], which have reported male preponderance with 72.9% and 72.35% being male, respectively. Male predominance in our study might be because alcohol intake, a major risk factor, is usually higher in males as compared to females.

Patients within 21-40 years of age were the most affected by AP to the tune of 70% (42/60) in our study. This is comparable to Vengadakrishnan et al.’s study with a reported identical age group (21-40 years) preponderance (49.09%) [7].

Gallstones and ethanol predominate while hypertriglyceridemia, drugs, and endoscopic retrograde cholangiopancreatography (ERCP) are notable among many causes [1]. The etiology of AP should be determined in at least 80% of cases, and no more than 20% should be classified as idiopathic [4]. Alcohol consumption, a modifiable risk factor for pancreatitis, is linked with the development of both acute and chronic pancreatitis. Moreover, this association between alcohol consumption and pancreatitis is biologically plausible; however, the underlying dose-response relationship remains poorly quantified. Ethanol has numerous deleterious effects on the pancreas, including direct toxicity to pancreatic cells. Mechanical obstruction of pancreatic ducts and pancreatic autodigestion; pancreato-toxic effects of ethanol metabolites (e.g., acetaldehyde, reactive oxygen species, fatty acid ethyl esters) and pancreato-fibrosis are various pathophysiological pathways that synergically contribute to alcohol-induced pancreatitis [8]. We have observed that alcohol is the most common etiological factor in 76.7% of patients, and this is comparable to the studies done by Negi et al. [6] and Rao et al. [9], where it was 59.3% and 51%, respectively. A very small number of female participants in our study (i.e., seven females as compared to 53 males) is the most probable explanation for alcohol being the most common etiological factor in our study.

There is a statistically significant difference (p<0.001) in the etiological factors of AP between males and females with alcohol being the most common etiology in males (86.7%) and cholelithiasis in females (71.4%) in our study. This observation correlates with studies by Jha et al. [10] where alcohol was the most common etiology in males (78%) and gallstones in females (88%) as well as the study by Reid et al. [11], where it is reported that alcoholic pancreatitis only occurred in males.

Pain abdomen was the most common clinical feature, as it was present in 96.7% (58/60) of patients, followed by vomiting in 65% (39/60) of patients. The clinical presentation in our study correlates with studies by Kumar et al. [12], where all the patients (100%) presented with pain abdomen, followed by 84% presented with nausea/vomiting, and a study by Chauhan et al. [13], where abdominal pain was present in 100% of the patients. In our study, there was no significant difference in the clinical features of AP between males and females.

Local complications of AP are acute peri-pancreatic fluid collection (APFC), pancreatic pseudocyst, acute necrotic collection (ANC), infected pancreatic necrosis, and walled-off necrosis (WON) [3,7,13]. Moreover, we have observed that among pancreatic complications, acute fluid collection was the most common complication seen in 26.7% (16/60) patients. This is comparable to the studies done by Ramu et al. [5] and Akhter et al. [14], where acute fluid collection was the most common pancreatic complication (29.1% and 41% respectively) reported by both studies.

Extra-pancreatic/systemic complications of AP include pulmonary, cardiovascular, hematological, gastrointestinal, renal, metabolic, and central nervous system (CNS) complications. The most common extra-pancreatic complication noted in our study was ascites seen in 50% of the patients. Pleural effusion (15%), shock (15%), MODS (15%), and hypocalcemia (11.7%) were the other extra-pancreatic complications observed. Our extra-pancreatic complications results are comparable to a study by Vengadakrishnan et al. [7], which reported that out of 110 patients, 20 patients (18.2%) had MODS, 15 patients (13.6%) had pleural effusion, nine patients (8.2%) had pseudocyst, two patients (1.8%) had hypotension, two patients (1.8%) had acute respiratory distress syndrome (ARDS), and two patients (1.8%) had diabetic ketoacidosis (DKA).

The decreased mean value of serum albumin and low platelet count were observed in the complications group as compared to the no-complications group in our study, and this difference was statistically significant (P<0.05). Our observations are comparable to a study by Xu et al. [15], where decreased serum albumin was noted as an independent predictor for severe AP and in-hospital mortality in AP patients. Lower serum albumin is related to decreased oncotic pressure, third space fluid loss, and, hence, higher complications. The lower mean platelet count reported in our study could be a part of MODS cases or maybe because of alcohol-induced toxicity.

Differences in the mean values of serum calcium, blood urea, serum creatinine, and serum amylase were not statistically significant between the complications group and the no-complications group in our study. Increased mean values of serum lipase and serum total bilirubin were observed in the complications group as compared to the no-complications group. This increase in serum lipase and serum bilirubin levels was significantly associated (P<0.05) with an increase in pancreatic/extra-pancreatic complications (Table no. 3); our observation is comparable to Vengadakrishnan et al.’s [7] study, where they have reported that raised serum lipase level is correlated well with poorer outcome.

Limitations of the study

The limitations of our study are the small sample size and a cross-sectional study conducted at a single center. Thus, a multicentric prospective study with a larger sample size can be planned in light of the present study to provide further information about the role of these studied factors as predictors of disease progression in AP.

## Conclusions

AP should be one of the differentials for patients presenting with pain abdomen, especially when probable risk factors like alcohol abuse and cholelithiasis are present. A high index of suspicion to diagnose APis needed because timely management may prevent systematic complications, thus improving the outcome. Poor prognostic indicators are raised levels of total serum bilirubin, raised serum lipase, reduced serum albumin, and low platelet count among AP patients. However, more studies are required especially in the Indian context to further identify the remediable factors that can improve the progression of the disease.
